# Four-dimensional left atrial quantification for predicting major adverse cardiovascular events in patients with uremia: an interpretable LASSO-logistic model

**DOI:** 10.3389/fendo.2026.1970519

**Published:** 2026-10-30

**Authors:** Lingxiang Ma, Mei Jin, Zikang Lin, Ruman Cui, Xuning Huang, Meihua Chen

**Affiliations:** Department of Ultrasound Medicine, The Second Affiliated Hospital of Hainan Medical University, Haikou, China

**Keywords:** four−dimensional left atrial quantification, machine learning, major adverse cardiovascular events, SHAP, uremia

## Abstract

**Background:**

This study aimed to develop a machine learning model using four-dimensional left atrial quantification (4D-LAQ) parameters to the risk of major adverse cardiovascular events (MACE) in patients with uremia.

**Methods:**

A total of 223 patients with uremia treated at the Second Affiliated Hospital of Hainan Medical University between January 2021 and January 2026 were retrospectively enrolled and followed up for the occurrence of MACE. LASSO regression was utilized for feature selection, followed by the development of eight prediction models. Logistic regression (LR) was identified as the final algorithm based on DeLong’s test. A nomogram was constructed, and Shapley Additive Explanations (SHAP) analysis was performed to enhance model interpretability. Model performance was comprehensively evaluated using receiver operating characteristic (ROC) curves, calibration plots, and decision curve analysis (DCA). Intra- and inter-observer reproducibility were assessed using the intraclass correlation coefficient (ICC).

**Results:**

During the follow-up period, 94 patients (42.1%) developed MACE. The MACE group exhibited left ventricular remodeling, mainly characterized by left atrial volume enlargement and a decrease in strain. The LR model achieved an AUC of 0.840 (95% CI: 0.749–0.931) in the validation set. Multiple validation approaches, including five-fold cross-validation, nested 5×5 cross-validation, and bootstrap internal validation, yielded AUCs of 0.873, 0.856, and 0.869, respectively. The calibration analysis showed good consistency (Hosmer-Lemeshow test, P > 0.05). The AUC of DCA (AUDC) was 0.208 (0.206 - 0.208), and the maximum net benefit was 0.43. The optimal risk stratification threshold, determined by the maximum Youden index in the training set, was 0.41. SHAP analysis identified left atrial reservoir strain (LASr) as the most influential predictive feature in this cohort.

**Conclusion:**

The LASSO-LR model integrating 4D-LAQ parameters may help MACE risk in patients with uremia, with LASr emerging as an important predictor.

## Graphical Abstract

**Figure:**
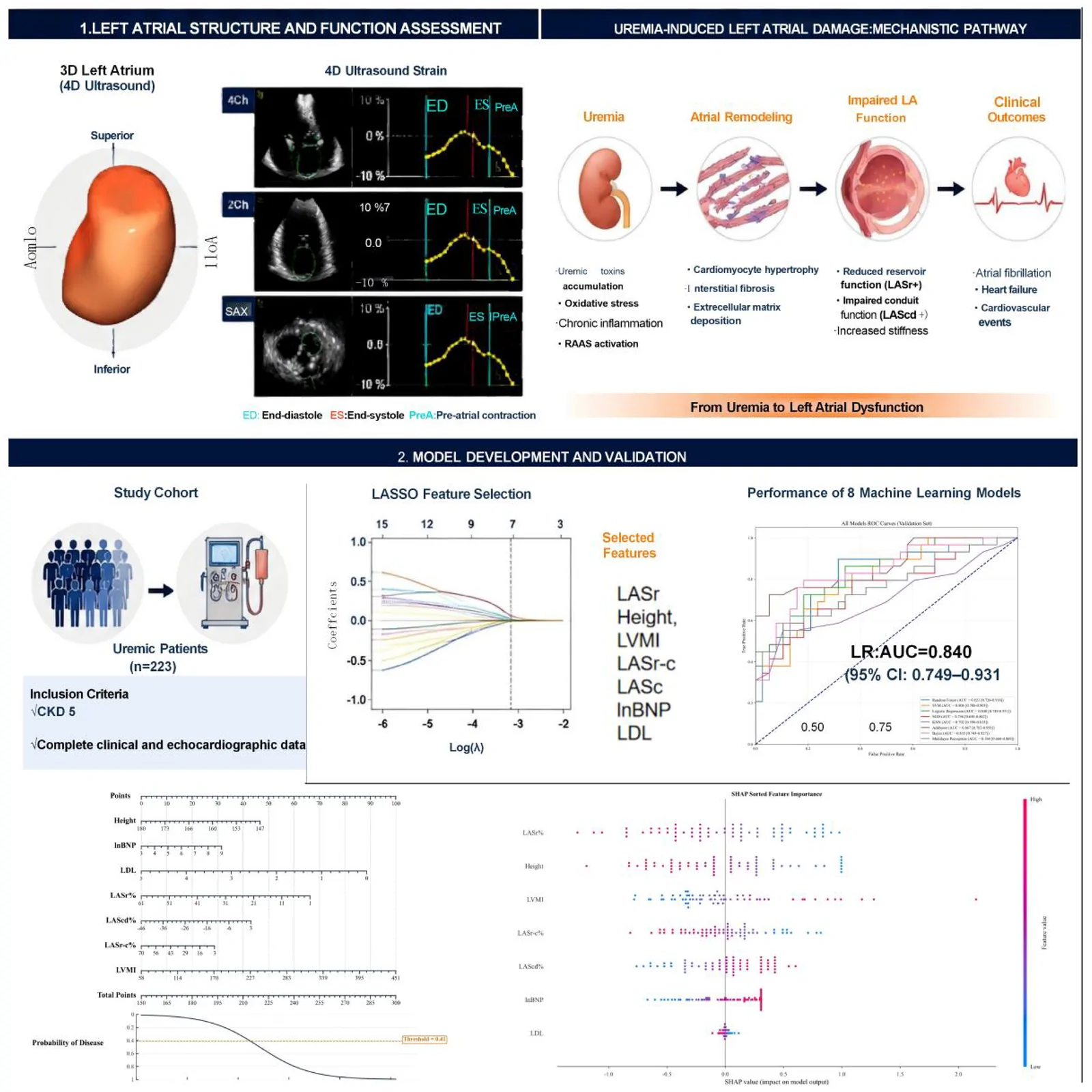

## Introduction

Chronic kidney disease (CKD) is a critical driver of rising morbidity and mortality attributable to noncommunicable diseases. According to the Global Burden of Disease Study, CKD ranked among the top ten leading contributors to the global disease burden between 1990 and 2019 (1, 2). In China, the rising prevalence of CKD, particularly in its advanced stages, poses an escalating challenge to the public health system. Among the dire consequences of CKD progression, cardiovascular disease (CVD) remains the leading cause of death, accounting for approximately 40% to 50% of mortality in patients with CKD (3, 4). This is because multiple mechanisms, including toxin accumulation, excessive neurohormone activation, and mineral bone metabolism disorders, lead to changes in the structure and function of the left ventricle, significantly increasing the risk of cardiovascular events (5, 6). These cardiovascular events not only prolong hospital stays and escalate treatment costs, but also compromise patients’ quality of life through cardio-renal interactions.

Echocardiography is the preferred imaging modality for evaluating cardiac involvement in patients with uremia because it is non-invasive, relatively inexpensive, and provides dynamic, repeated assessment of cardiac structure and function (7). Increasing evidence indicates that, in patients with uremia, left atrial remodeling often precedes the development of left ventricular dysfunction, suggesting that left atrial assessment may aid in the early prediction of prognosis (8). 4D-LAQ is used to assess the volume and strain of the left atrium, thereby enabling simple, rapid quantification of its structure and function (9). This technique has been applied in the diagnosis of conditions such as coronary heart disease and atrial fibrillation (9, 10). However, the predictive value of 4D-LAQ parameters for MACE in patients with uremia remains unclear. Therefore, this study aims to establish a model based on 4D-LAQ parameters to predict the risk of MACE in patients with uremia.

The traditional LR model often struggles to capture the inherent nonlinear relationships and complex interaction effects in high-dimensional clinical data, and may be limited by multicollinearity and overfitting (11). Combining LASSO’s penalized regression and comparative modeling framework helps overcome these limitations (12). Moreover, the rapid development of computational science has enabled machine learning (ML) to be widely applied in medical prognosis research in recent years. More and more medical prognosis studies adopt an analytical framework that combines penalized regression, machine learning algorithms, and interpretability methods (11, 13). These methods can systematically compare multiple candidate models and identify the predictors related to clinical outcomes. However, the selection of the final model should be based on discrimination, calibration, clinical benefits, and overfitting risk, rather than just model complexity.

Recent breakthroughs in explainable artificial intelligence have enabled the decoding of machine learning models. Among these, the SHAP method has emerged as one of the most widely adopted interpretability analysis tools (14). SHAP quantifies the marginal contribution of each feature to the model’s predictions, revealing both global feature importance rankings and local explanations for individual predictions (14, 15). By decomposing the model’s predictions into feature-specific contribution values, SHAP effectively enhances the transparency and interpretability of complex models, thereby facilitating the clinical application and acceptance of machine learning in practice (14, 16).

## Materials and methods

### Study population

Patients with uremia treated at the Second Affiliated Hospital of Hainan Medical University between January 2021 and January 2026 were enrolled. The index date for each participant was the date of confirmed diagnosis of uremia. Each participant was followed from the baseline date until March 2026. If the MACE occurred during follow-up, the date of the event was recorded, and follow-up was discontinued at that point. Participants who did not experience MACE were excluded in March 2026. The inclusion criteria were as follows: (1) diagnosis of CKD stage 5 according to the 2024 KDIGO Clinical Practice Guideline for the Evaluation and Management of Chronic Kidney Disease (17), with an estimated glomerular filtration rate (eGFR) < 15 mL/min/1.73 m²; and (2) Availability of baseline clinical, laboratory, and echocardiographic data. The exclusion criteria were as follows: (1) poor image quality; (2) congenital heart disease (atrial septal defect, ventricular septal defect, patent ductus arteriosus, tetralogy of Fallot); (3) moderate-to-severe valvular stenosis or regurgitation of any etiology; (4) arrhythmias due to other causes; (5) prior kidney transplantation; and (6) inability to provide informed consent due to psychiatric disorders. A patient screening and modeling flowchart is presented in **Figure 1**.

**Figure 1:**
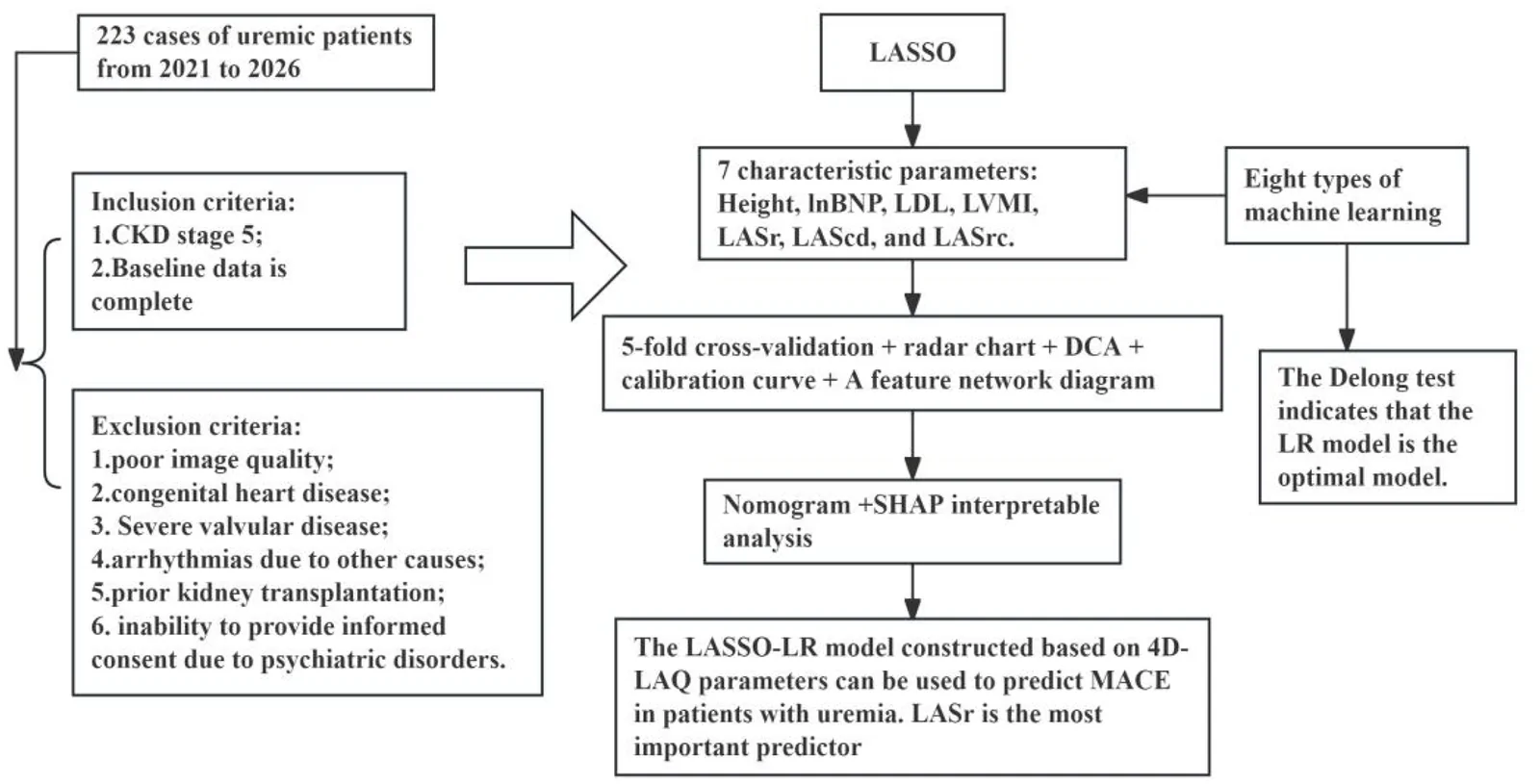
Flowchart.

This retrospective cohort study was approved by the Ethics Committee of the Second Affiliated Hospital of Hainan Medical University (Ethics Number: 2026-K10-01). All patients signed an informed consent form. This study was conducted in accordance with the Declaration of Helsinki.

### Instruments and methods

Baseline data for all patients were collected from the hospital electronic medical record system, including age, sex, height, weight, and body surface area (BSA). Laboratory indicators included serum creatinine, blood urea nitrogen, B-type natriuretic peptide (BNP), eGFR, hemoglobin (HB), red blood cell count (RBC), serum calcium, serum phosphorus, total cholesterol, triglycerides, low-density lipoprotein cholesterol (LDL), high-density lipoprotein cholesterol (HDL), and parathyroid hormone. BNP values exhibited an extremely right-skewed distribution and were therefore natural log-transformed (lnBNP) before modeling to mitigate the influence of extreme values and to stabilize regression coefficients.

Echocardiographic examinations were performed using a GE Vivid E95 ultrasound system equipped with M5Sc (frequency, 1.4–4.6 MHz) and 4Vc (frequency, 1.4–5.2 MHz) transducers, with offline analysis conducted on a GE EchoPAC PC 203 workstation. Left ventricular parasternal long-axis views were obtained to measure the left atrial anteroposterior diameter (LAD), interventricular septal thickness (IVS), left ventricular posterior wall thickness (LVPWT), and left ventricular end-diastolic diameter (LVEDD). Left ventricular ejection fraction (LVEF) was measured using the modified biplane Simpson method. Dynamic images were imported into the EchoPAC PC 203 workstation, and the left atrial endocardium was automatically traced using the system software, with manual adjustment when necessary, to obtain satisfactory region of interest data. The following 4D-LAQ parameters were derived: LAVmin, LAVmax, LAVpreA, LAVImax, LAEV, LAEF, LASr, LAScd, LASct, LASr-c, LAScd-c, and LASct-c (full names are provided in **Supplementary Table 1**).

All images were acquired by the same ultrasonographer with more than five years of experience in diagnosis. The data were repeated three times by two other experienced doctors, and the average was calculated.

### Feature selection and model selection

This study estimates the minimum sample size based on the event number per variable (EPV) criterion. The 223 patients were randomly stratified into training (n = 156) and validation (n = 67) sets in a 7:3 ratio. The training set was used for hyperparameter optimization, and the internal validation set was used for performance evaluation. An additional 5-fold stratified cross-validation was performed to further assess model stability, with each fold maintaining the MACE event proportion consistent with the original cohort.

LASSO-penalized logistic regression with binomial deviance was applied to screen all candidate predictors, with the optimal regularization parameter α determined using the minimum deviance (min λ) and 1-standard-error (1-SE λ) criteria via cross-validation. Pearson correlation analysis and Mantel’s test were conducted to evaluate multicollinearity among the selected features.

Based on the characteristic parameters, eight common prediction models were constructed: RF, SVM, LR, SGD, KNN, AdaBoost, Bayes, and MLP. The performance of the final model was visualized using a radar chart, covering the following indicators: area under the curve (AUC), accuracy, sensitivity, specificity, precision, negative predictive value (NPV), recall rate, and F1 score. The discriminative ability and clinical practicability of the model were evaluated using DCA, and the model fitting was tested using calibration curves. A nomogram for individualized risk quantification was constructed based on the LR model. The optimal stratification threshold was defined as the point that maximized the Youden index on the ROC curve. Patients were divided into high- and low-risk groups based on the optimal probability threshold. SHAP was used to explain feature importance, and a feature network diagram was constructed to verify data consistency among all statistical modules.

### Outcomes

MACE was confirmed through the electronic medical record system and telephone follow-up. MACE was defined as a composite of cardiac death, hospitalization for worsening heart failure, non-fatal stroke, and non-fatal myocardial infarction (3). The individual components of the MACE were as follows: cardiac death, n = 0; hospitalization for worsening heart failure, n = 54; non-fatal stroke, n = 26; and non-fatal myocardial infarction, n = 14. No loss to follow-up occurred. By reverse Kaplan-Meier analysis, the median follow-up period was 20 months (interquartile range: 17–48 months), and the average follow-up period was 30 months.

### Statistical analysis

Statistical analyses and data visualizations were performed using R (version 4.6.1) and SPSS 27. Normally distributed continuous variables are presented as mean ± standard deviation (SD) and compared using independent Student’s t-tests. Non-normally distributed continuous variables are presented as medians (interquartile range, IQR) and were compared using the Mann–Whitney U test. Categorical variables were compared using the chi-square test. Highly skewed BNP values were subjected to natural logarithmic transformation (lnBNP) to mitigate skewness and stabilize the variance before model construction. A two-sided P-value < 0.05 was considered statistically significant. Variables with missing data exceeding 20% were excluded. The overall proportion of missing data in the dataset is 4.2%. The variables with the highest missing rate are HDL (28 cases, 12.5%) and LDL (30 cases, 13.4%). Among the remaining variables in the study, 34% (13 out of 38 variables) had missing values, which were filled using the multiple imputation method.

For machine learning modeling, all random processes were controlled using a fixed random seed (seed = 42) to ensure reproducibility. Hyperparameter tuning was performed using randomized search with 5-fold stratified cross-validation, using the area under the ROC-AUC as the primary scoring metric. Tuning was conducted exclusively on the training set, and the validation set was held out for performance evaluation. The hyperparameter search spaces and final selected hyperparameters for all eight machine learning models are provided in **Supplementary Figure 1**, **Supplementary Table 2**.

## Results

### Baseline characteristics

As shown in **Table 1**, 223 patients with uremia were included, of whom 94 (42.1%) experienced MACE during follow-up. Compared with the non-MACE group, the MACE group showed significantly increased age, LAD, IVS, LVEDD, LVPWT, LVMI, BNP, LAVmin, LAVmax, LAVpreA, and LAVImax (all P < 0.05), while the absolute values of Height, RBC, LDL, LAEF, LASr, LAScd, LASct, LASr-c, LAScd-c and LASct-c were significantly decreased (all P < 0.05). Additionally, the proportion of male patients in the MACE group (77.7%) was significantly higher than that in the non-MACE group (54.3%, P < 0.05). These intergroup differences suggest that patients who develop MACE have more severe left ventricular hypertrophy and left atrial functional impairment.

**Table 1:** Baseline characteristics according to MACE occurrence.

Variables	Total (n = 223)	N-MACE (n = 129)	MACE (n = 94)	P-value
Gender, n (%)				<0.001
Female	80 (35.9)	59 (45.7)	21 (22.3)	
Male	143 (64.1)	70 (54.3)	73 (77.7)	
Age, years	47.0 (37.0, 57.0)	46.0 (36.0, 54.0)	50.5 (38.0, 59.0)	0.033
Height, cm	164.0 (158.0, 169.5)	165.0 (160.0, 170.0)	162.0 (156.2, 168.0)	0.042
Weight, kg	58.0 (51.5, 65.0)	59.0 (52.0, 66.0)	57.2 (51.0, 62.0)	0.313
BSA, m²	1.6 ± 0.2	1.6 ± 0.2	1.6 ± 0.2	0.203
SBP, mmHg	146.2 ± 22.1	143.9 ± 19.7	149.5 ± 24.7	0.062
DBP, mmHg	88.7 ± 13.3	88.3 ± 13.2	89.3 ± 13.3	0.570
LAD, mm	37.0 (32.0, 40.0)	35.0 (31.0, 38.0)	39.0 (35.0, 42.0)	<0.001
IVS, mm	13.0 (12.0, 14.0)	12.0 (11.0, 13.0)	13.0 (12.0, 15.0)	<0.001
LVEDD, mm	46.5 ± 6.5	45.0 ± 5.7	48.5 ± 7.0	<0.001
LVPWT, mm	12.0 (11.0, 13.5)	12.0 (11.0, 13.0)	13.0 (12.0, 14.0)	<0.001
LVEF, %	63.0 (58.0, 68.0)	64.0 (59.0, 68.0)	62.0 (56.0, 67.0)	0.056
Creatinine, μmol/L	994.0 (753.5, 1316.0)	952.0 (724.0, 1294.0)	1062.0 (779.5, 1329.0)	0.162
BUN, mmol/L	22.5 (18.4, 28.8)	22.0 (17.5, 28.0)	23.1 (19.4, 29.2)	0.214
BNP, pg/mL	5658.0 (1312.5, 15098.6)	3902.0 (808.0, 8541.8)	12353.7 (2827.5, 29431.0)	<0.001
eGFR, ml/(min*1.73m^2^)	11.0 (4.0, 16.0)	11.0 (5.0, 16.0)	10.5 (4.0, 15.8)	0.302
Hb, g/L	101.2 ± 25.4	103.9 ± 23.1	97.4 ± 27.9	0.056
RBC, 10^12^/L	3.6 (3.0, 4.2)	3.7 (3.2, 4.3)	3.4 (2.7, 4.0)	0.028
Calcium, mmol/L	2.3 (2.1, 2.4)	2.2 (2.1, 2.4)	2.3 (2.1, 2.4)	0.143
Phosphorus, mmol/L	1.8 (1.4, 2.4)	1.9 (1.3, 2.5)	1.7 (1.4, 2.3)	0.512
Cholesterol, mmol/L	4.0 (3.2, 4.6)	4.0 (3.5, 4.6)	3.8 (3.0, 4.6)	0.105
Parathyroid hormone, pg/mL	322.6 (154.8, 522.5)	309.2 (153.0, 458.8)	341.5 (160.3, 590.8)	0.209
Triglyceride, mmol/L	1.2 (0.8, 1.4)	1.2 (0.8, 1.4)	1.2 (0.8, 1.4)	0.437
LDL, mmol/L	2.4 (2.1, 2.7)	2.5 (2.2, 2.7)	2.2 (1.9, 2.8)	<0.001
HDL, mmol/L	1.2 (1.0, 1.3)	1.2 (1.0, 1.3)	1.2 (1.0, 1.4)	0.220
LAVmin, ml	25.0 (18.0, 32.0)	22.0 (17.0, 29.0)	28.0 (21.0, 39.5)	<0.001
LAVmax, ml	55.0 (43.0, 66.0)	54.0 (41.0, 63.0)	58.0 (47.0, 70.0)	0.009
LAVpreA, ml	41.0 (31.0, 51.0)	38.0 (29.0, 45.0)	46.0 (36.0, 57.8)	<0.001
LAVImax, ml/m²	35.0 (26.0, 40.5)	33.0 (24.0, 39.0)	37.0 (28.2, 42.8)	0.003
LAEV, ml	28.0 (22.5, 34.0)	29.0 (23.0, 34.0)	28.0 (22.2, 33.0)	0.595
LAEF, %	54.0 (47.0, 59.5)	56.0 (52.0, 61.0)	49.0 (44.0, 57.0)	<0.001
LASr, %	18.0 (13.0, 24.0)	22.0 (17.0, 28.0)	13.0 (8.0, 19.0)	<0.001
LAScd, %	-9.0 (-14.0, -6.0)	-12.0 (-15.0, -7.0)	-6.0 (-10.0, -2.0)	<0.001
LASct, %	-9.0 (-13.0, -5.0)	-11.0 (-14.0, -6.0)	-7.0 (-11.8, -4.0)	<0.001
LASr-c, %	30.0 (23.0, 36.0)	32.0 (26.0, 40.0)	25.0 (19.0, 31.0)	<0.001
LAScd-c, %	-12.0 (-17.0, -7.0)	-14.0 (-20.0, -9.0)	-9.0 (-15.0, -4.2)	<0.001
LASct-c, %	-17.5 ± 8.5	-19.0 ± 8.4	-15.5 ± 8.2	0.002
LVMI, g/m²	136.7 (112.2, 161.1)	124.3 (105.7, 146.9)	156.3 (127.4, 190.4)	<0.001

Abbreviations are defined in the text. All categorical variables were described using the number of cases (percentage), statistical methods were described using the chi-square test or exact probability method, continuous variables were described using median (percentile) or mean ± standard deviation, and statistical tests were performed using non-parametric or t-tests. Statistical significance was set at P < 0.05. MACE, major adverse cardiovascular events; other abbreviations are as defined in **Supplementary Table 1**.

### Intraobserver and interobserver variability

The intra-observer intraclass correlation coefficients (ICCs) for LAVmin, LAVmax, LAVpreA, LAVImax, LAEV, LAEF, LASr, LAScd, LASct, LASr-c, LAScd-c, and LASct-c were 0.952, 0.923, 0.957, 0.932, 0.838, 0.864, 0.877, 0.841, 0.810, 0.875, 0.791, and 0.855, respectively. The corresponding inter-observer ICC values were 0.967, 0.935, 0.970, 0.904, 0.901, 0.813, 0.915, 0.841, 0.802, 0.915, 0.807, and 0.860, respectively. These findings indicate that LA strain measurements demonstrate satisfactory intra-and interobserver reproducibility (See **Table 2**).

**Table 2:** Intraobserver and interobserver variability.

Parameter	Intraobserver			Interobserver		
	ICC	95% CI	P value	ICC	95% CI	P value
LAVmin, ml	0.952	0.902-0.977	<0.001	0.967	0.931-0.984	<0.001
LAVmax, ml	0.923	0.846-0.963	<0.001	0.935	0.868-0.969	<0.001
LAVpreA, ml	0.957	0.913-0.979	<0.001	0.970	0.939-0.986	<0.001
LAVImax, ml/m²	0.932	0.862-0.967	<0.001	0.904	0.809-0.953	<0.001
LAEV, ml	0.838	0.687-0.919	<0.001	0.901	0.802-0.951	<0.001
LAEF, %	0.864	0.714-0.935	<0.001	0.813	0.644-0.906	<0.001
LASr, %	0.877	0.758-0.940	<0.001	0.949	0.896-0.975	<0.001
LAScd, %	0.841	0.666-0.924	<0.001	0.841	0.665-0.924	<0.001
LASct, %	0.810	0.638-0.905	<0.001	0.802	0.626-0.901	<0.001
LASr-c, %	0.875	0.755-0.939	<0.001	0.915	0.829-0.958	<0.001
LAScd-c, %	0.791	0.606-0.895	<0.001	0.807	0.634-0.903	<0.001
LASct-c, %	0.855	0.717-0.928	<0.001	0.860	0.727-0.931	<0.001

95% CI, 95% confidence interval; ICC, intraclass correlation coefficient.

### Feature selection using LASSO regression

To avoid multicollinearity and identify independent predictors, LASSO-penalized logistic regression was performed to select the variables. The regularization strength determined by the minimum cross-validated binomial deviance (α_min) and the optimal α value selected according to the 1-SE criterion both converged at α = 10 (**Figure 2A**), indicating that the sparse model obtained under the 1-SE criterion achieved cross-validation errors close to the minimum without significantly compromising predictive performance. The coefficient path plot visually depicts the shrinkage process of each feature at α = 10 and the variable selection results (**Figure 2B**). Ultimately, seven features with nonzero regression coefficients were identified as predictors of MACE in patients with uremia: height, lnBNP, LDL, LVMI, LASr, LAScd, and LASr-c.

**Figure 2:**
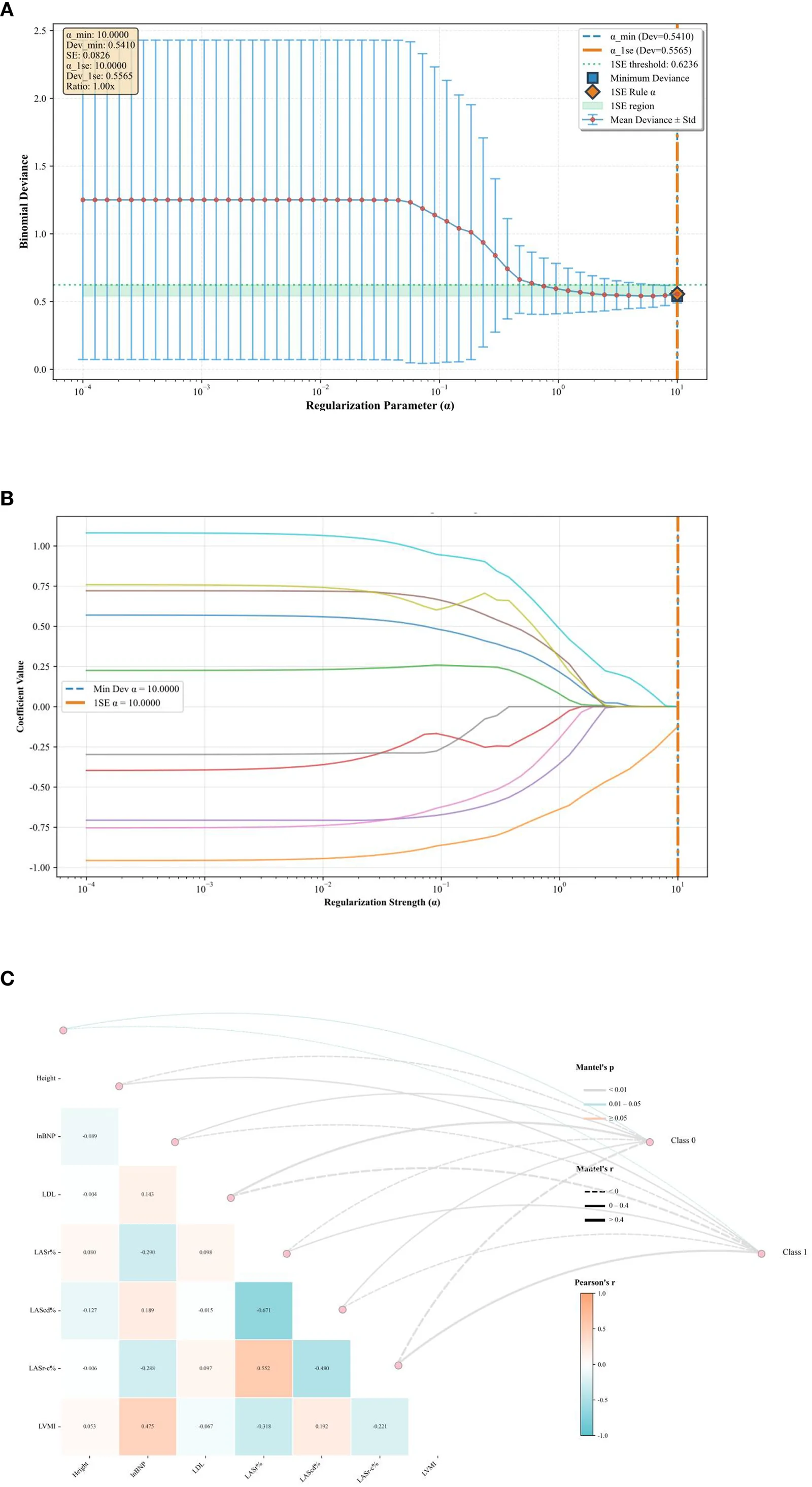
**(A)** Cross-validation binomial deviance curve. Cross-validation binomials. The square and diamond markers at α=10 represent the minimum deviance point and the 1SE optimal α, respectively. The text box summarizes the key cross-validation metrics for α_min and α_1se. **(B)** Coefficient path plot of all candidate predictors. Coefficient path plot of all the candidate predictors. Each colored line represents the regression coefficient of a single feature as it varies with regularization strength α. Features with coefficients that shrank to zero at α=10 were excluded from the final model. **(C)** Correlation Analysis of Predictive Indicators via Pearson and Mantel’s Test. Pearson correlation matrix and Mantel’s test linking candidate predictors to adverse cardiovascular events (class 1) and non-event controls (class 0).

Pearson’s correlation analysis and Mantel’s test on the seven selected variables (**Figure 2C**) demonstrated generally low correlation coefficients among the variables, with no strongly correlated pairs.

### Construction and performance evaluation of the LR model

Based on the selected features, eight commonly used prediction models were constructed. **Figure 3** shows the ROC, DCA, and calibration curves of each model in the training and validation sets. According to the DeLong’s test results (**Table 3**), the LR model was selected for subsequent development.

**Figure 3:**
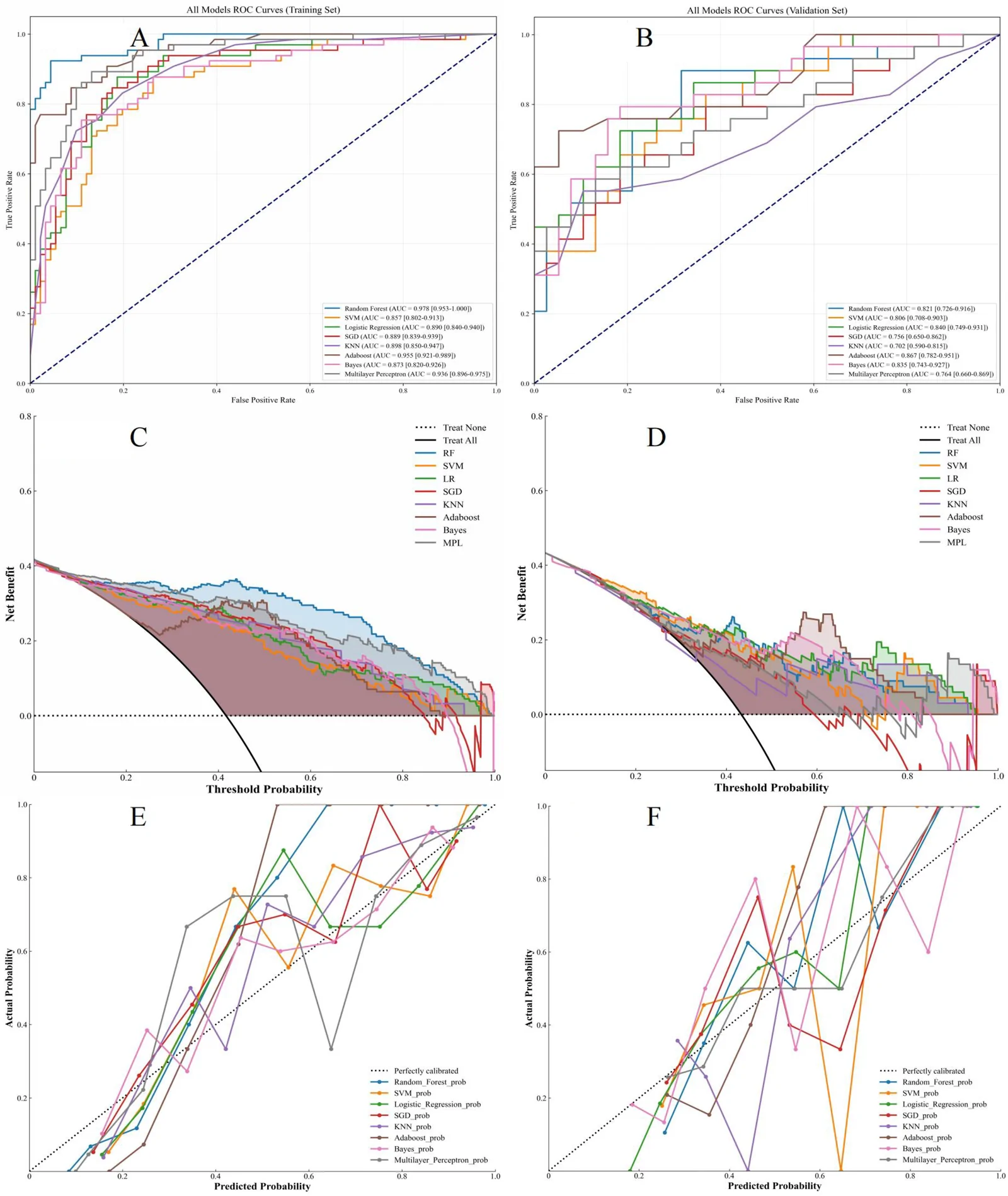
The ROC, DCA, and calibration curves of each model in the training and validation sets. **(A)** ROC curve of the training set; **(B)** ROC curve of the validation set. **(C)** Decision curve analysis of the training set; **(D)** Decision curve analysis of the validation set; **(E)** Calibration curve of the training set; **(F)** Calibration curve of the validation set.

**Table 3:** DeLong's test for each model.

Comparison	AUC1	AUC2	Z_score	NRI	IDI	P_value
LR vs Bayes	0.840	0.835	0.24	0.07	0.15	0.81
LR vs AdaBoost	0.840	0.867	-0.52	0.13	-0.09	0.60
LR vs SVM	0.840	0.806	-2.52	0.07	-0.01	0.01
LR vs RF	0.840	0.821	-0.47	-0.04	0.05	0.64
LR vs SGD	0.840	0.756	3.21	-0.1	-0.01	0.01
LR vs KNN	0.840	0.702	3.18	-0.09	-0.10	0.01
LR vs MLP	0.840	0.764	2.37	-0.08	-0.01	0.02

AUC, area under the receiver operating characteristic curve; CI, confidence interval; IDI, integrated discrimination improvement; NRI, net reclassification improvement.

**Figures 3A, B** showed that the AUC of the LR model in the training set was 0.890 (95% CI: 0.840–0.940), and in the validation set, it was 0.840 (95% CI: 0.749–0.931). The DCA curves (**Figures 3C, D**) indicated that the AUDC of the LR model in the training set was 0.215 (0.213 - 0.215), with the maximum net benefit was 0.41. In the validation set, the AUDC was 0.208 (0.206 - 0.208), and the maximum net benefit was 0.43 (**Supplementary Table 7**). The calibration curves (**Figures 3E, F**) were consistent with the ideal calibration diagonal in both groups, and the Hosmer-Lemeshow test P > 0.05 (**Supplementary Table 6**). **Figure 4** presented a radar chart of the LR model, including indicators such as sensitivity, specificity, and accuracy.

**Figure 4:**
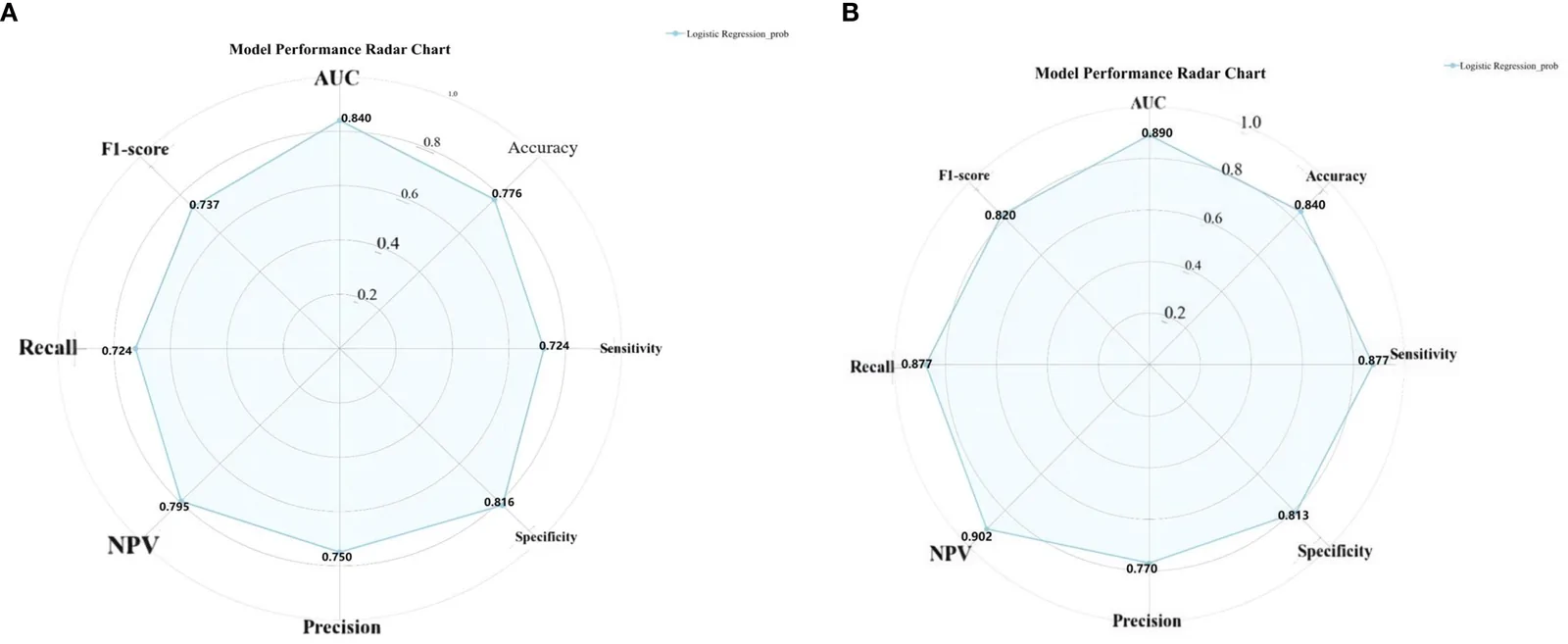
Radar chart of the LR model. **(A)** Radar plot of the training set. **(B)** Radar plot of the validation set. The radar plot includes the AUC values, Youden index, F1 score, recall rate, negative predictive value, positive predictive value, specificity, sensitivity and accuracy of the training set and validation set of the LR model.

This study employed a comprehensive set of internal validation strategies, including five-fold stratified cross-validation, nested 5×5 cross-validation, and bootstrap internal validation. The results of the five-fold stratified cross-validation (**Supplementary Table 3**) showed that the average AUC was 0.873 ± 0.053. The results of Nested 5×5 cross-validation (**Supplementary Table 4**) showed that the average AUC was 0.856 ± 0.020. The results of the bootstrap internal validation (B = 1000, **Supplementary Table 5**; **Supplementary Figure 2**) revealed an optimism-corrected AUC of 0.869, with a mean optimism of 0.025. The calibration slope was 1.000 (95% CI: 0.834–1.418), the Brier score was 0.132 (95% CI: 0.125–0.134), and the scaled Brier score was 0.458 (95% CI: 0.449–0.484). The bootstrap-corrected calibration curve approximated the ideal diagonal, indicating good calibration after optimism correction.

### The incremental value of 4D-LAQ

As shown in **Supplementary Table 8**, within the same validation framework, we constructed Model 1 (Clinical-only) and Model 2 (conventional echocardiography) to verify the incremental value of the original model (Model 3). The AUC, DCA and Calibration curves of the three preset models are shown in **Figure 5**. The results showed that on the validation set, the AUC of Model 1 was 0.703 (95% CI: 0.591–0.815), Model 2 was 0.750 (95% CI: 0.643–0.856), and Model 3 was 0.840 (95% CI: 0.749–0.931) (**Figure 5**). Compared with Models 1 and 2, the inclusion of 4D-LAQ significantly enhanced the discriminative ability of the model (vs Model 1, ΔAUC = 0.137, P < 0.05; vs Model 2, ΔAUC = 0.09, P < 0.05). Furthermore, Model 3 demonstrates superior DCA and calibration curves (**Supplementary Tables 6**, **7**).

**Figure 5:**
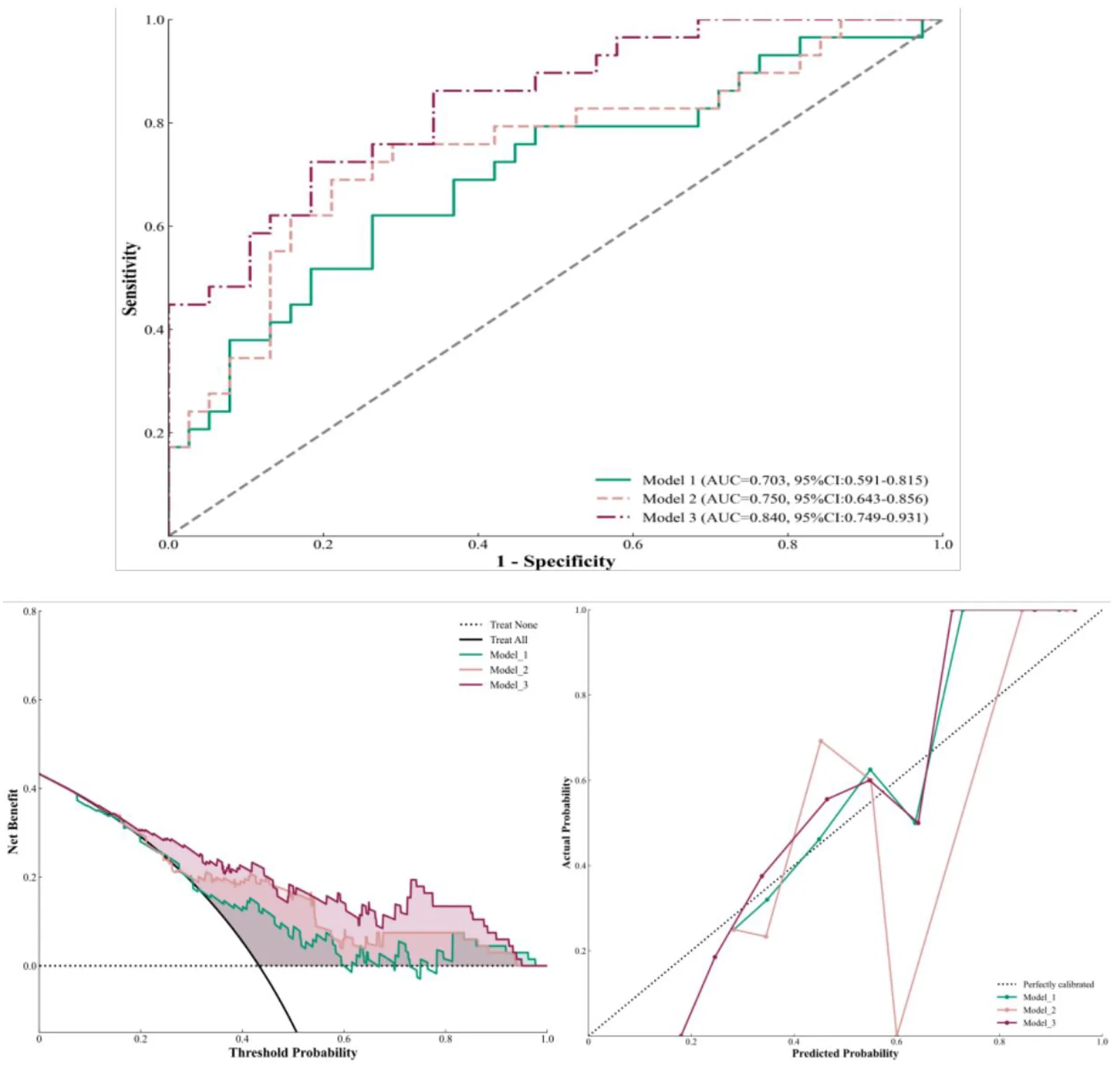
The ROC, DCA, and calibration curves of the prespecified models. The AUC, DCA and Calibration curves of the three preset models.

### Construction of the nomogram for the prediction model

A nomogram was constructed based on the seven features selected by the LASSO regression to quantify the individualized MACE risk (**Figure 6**). The cutoff value of 0.41 was determined as the optimal risk stratification threshold based on the maximum Youden index on the ROC curve in the training set. At this cutoff, the sensitivity and specificity were 87.7% and 81.3%, respectively, in the training set and 72.4% and 81.6%, respectively, in the validation set (**Figure 4**). Patients with a predicted probability > 0.41 were classified as high-risk for MACE, while those with a predicted probability < 0.41 were classified as low-risk.

**Figure 6:**
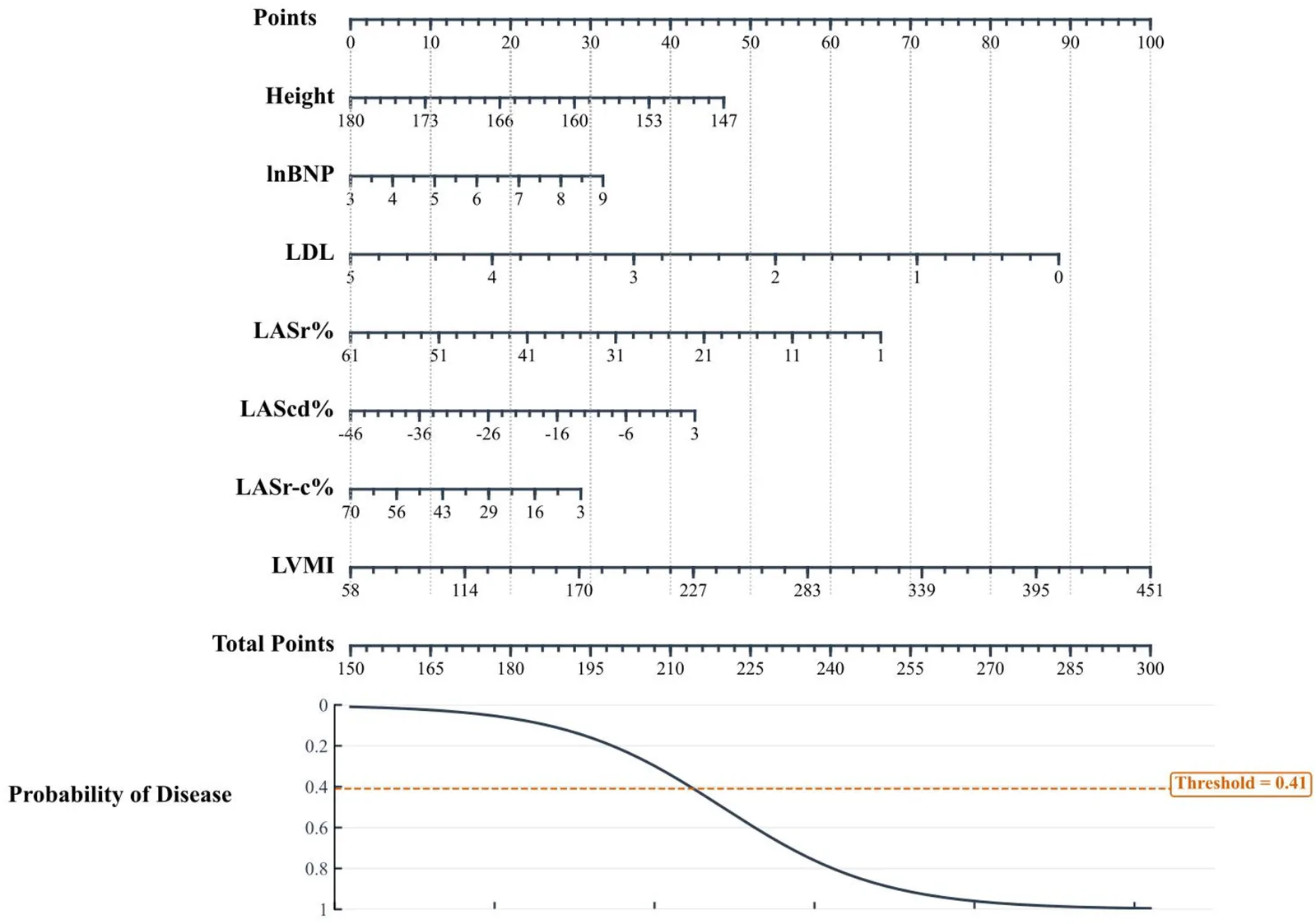
Construction of the Nomogram for the prediction model. For an individual patient, the point value for each variable was read from the uppermost “Points” axis. The sum of these points was then located on the “Total Points” axis, and the corresponding predicted probability was derived from the bottom of the risk scale. The orange dashed line marks the optimal risk threshold of the predicted probability at 0.41, which was determined by ROC curve analysis.

### SHAP explanation of the LR model

SHAP analysis was further applied to rank the feature importance. **Figure 7** displays the feature importance ranking, showing that the most influential parameters in the model were LASr, Height, LVMI, LASr-c, LAScd, lnBNP, and LDL. The scatter distribution in the figure reveals that lower LASr values corresponded to higher positive SHAP contributions, indicating increased MACE risk. lnBNP and LDL exhibited relatively small SHAP values, suggesting only marginal contributions to the model’s predictions.

**Figure 7:**
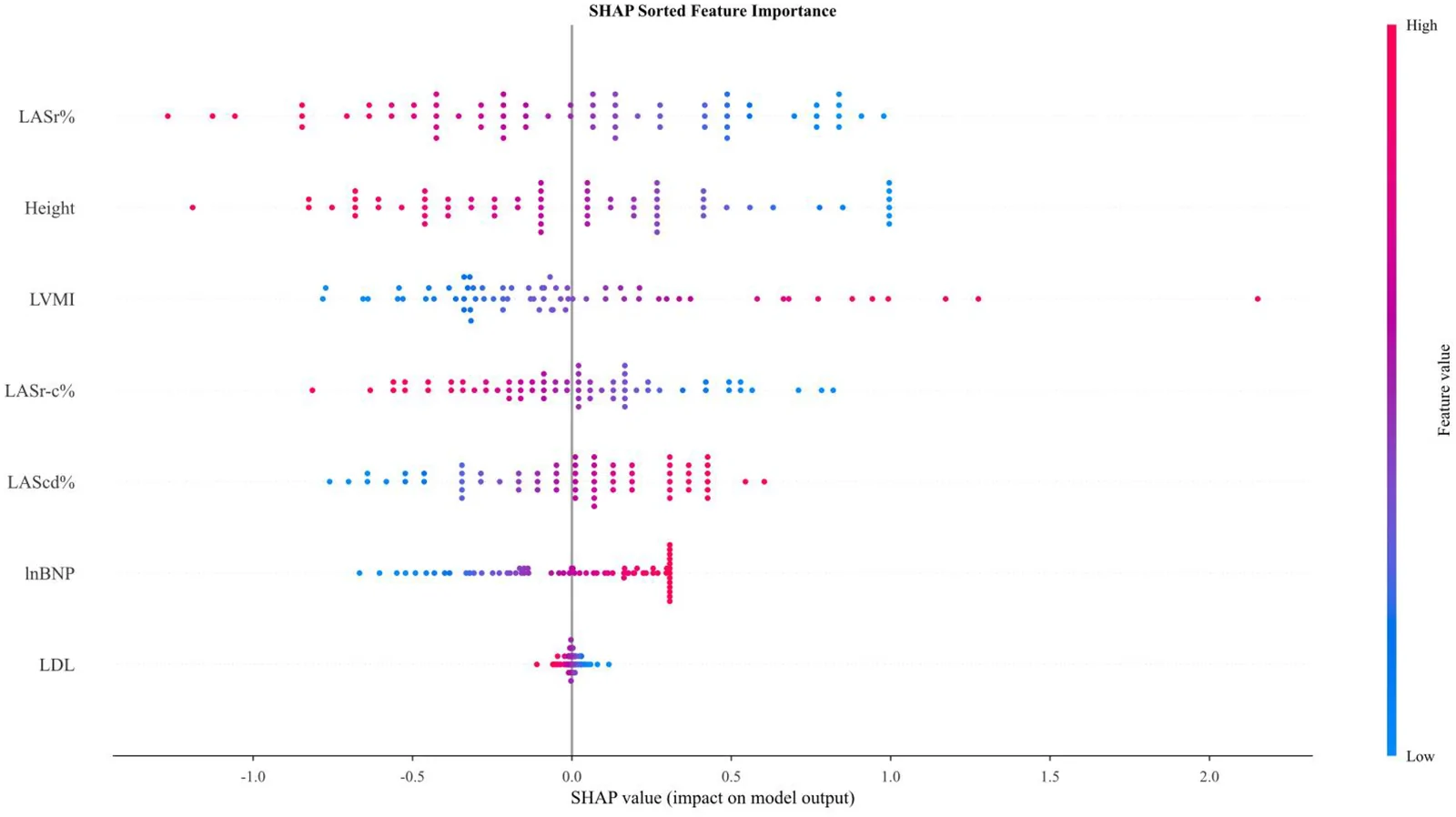
SHAP Sorted Feature Importance. Each point corresponds to the SHAP value of a single patient on a certain variable. The horizontal axis represents the SHAP value: positive values increase the probability of MACE prediction, while negative values decrease the MACE probability.

## Discussion

This study combined the 4D-LAQ parameters and constructed an interpretable machine learning prediction model that was validated internally. The main findings were as follows: (1) left atrial remodeling occurred in the MACE group, manifested as left atrial enlargement and decreased strain; (2) the seven most important features in the model were LASr, Height, LVMI, LASr-c, LAScd, lnBNP, and LDL; (3) the LR model demonstrated good discrimination in internal validation, along with favorable clinical net benefit and calibration; (4) a nomogram was constructed with an optimal risk stratification threshold of 0.41 determined by the Youden index; and (5) SHAP importance ranking identified LASr as the most influential predictor in this cohort, with reduced LASr strongly associated with increased MACE risk.

A large number of studies have shown that in patients with uremia, the prognostic value of the left atrium may be higher than that of the left ventricle. Its structure and function are closely related to diseases such as atrial fibrillation and heart failure (18, 19). Our research supports this view, as in patients with uremia who experienced MACE, the volume and strain parameters of the left atrium deteriorated further. Notably, all left atrial volume parameters were eliminated by LASSO regression, whereas only three left atrial strain parameters—LASr, LAScd, and LASr-c—were retained in the final model. This suggests that strain parameters have stronger discriminative efficacy than volume parameters in predicting MACE risk. By quantifying myocardial deformation, strain parameters sensitively capture changes in left atrial wall pressure and deformation, thereby enabling a comprehensive assessment of left atrial phasic function, which is typically divided into three phases: reservoir, conduit, and booster pump (20–22). These three phase reflect left atrial compliance, left ventricular suction capacity during early diastole, and intrinsic left atrial contractility (20–22). Consistent with previous studies (23, 24), patients with uremia who experienced MACE showed impaired left atrial strain. Our study corroborated these findings, showing that all six left atrial strain parameters (LASr, LAScd, LASct, LASr-c, LAScd-c, and LASct-c) were significantly decreased in the MACE group. In combination with the SHAP plot, we further discovered that impaired reservoir function is the core component of left atrial strain dysfunction, and LASr, which represents longitudinal reservoir function, is the most significant predictive factor in this model. Indeed, LASr integrates information on left ventricular systolic and diastolic function, chamber dimensions, and hemodynamics, and has demonstrated robust predictive value for adverse outcomes across diverse populations, outperforming traditional clinical risk scores and other imaging parameters (20, 25, 26).

Beyond left heart-related ultrasound parameters, LASSO regression also identified additional features: height, lnBNP, and LDL.Although Height ranked second in the SHAP summary plot, this finding should not be interpreted as an independent biological effect. Due to the mathematical coupling relationship between Height and LVMI, SHAP allocated some of the contributions to Height. This phenomenon should be explained as a statistical illusion caused by mathematical coupling and collinearity. BNP is a biomarker that has been proven to be a marker of myocardial wall stress and volume overload (27). Due to the extremely right-skewed distribution of BNP, the data in this study were stabilized through natural logarithmic transformation before being included in the model. In this study, the “lipid paradox” was observed for LDL (28); that is, a low LDL level was actually associated with a poor prognosis. This often indicates that the patient has a more severe state of catabolism, micro-inflammatory load, and protein-energy depletion, which jointly drive adverse cardiovascular outcomes (28, 29). Given that LDL occupied the lowest SHAP rank in our model, its weight in decision-making should not be overemphasized; clinicians applying this model should prioritize ultrasound parameters, such as LASr.

Combining DeLong’s test and the characteristics of the dataset, we finally selected the LR model as the final model. The overall discrimination performance of this model was superior to that of the cardiovascular event prediction model for patients with uremia that only included clinical indicators (30, 31). Although AdaBoost had a higher AUC (0.867) in the validation set, the AUC in the training set was 0.955, indicating a significant overfitting. Although DeLong’s test (**Table 3**) showed Bayes have discriminant performance comparable to logistic regression (LR) (AUC: 0.835 vs. 0.840, P = 0.807), Bayes on strict conditional independence assumptions, meaning that all features are independent of each other under the premise of a given category label (32), which does not match our dataset. In contrast, logistic regression does not require such assumptions and is therefore more robust in clinical settings. Additionally, in cases with small or medium sample sizes, the generalization performance of the LASSO-LR combination is usually superior to complex nonlinear models (33, 34). Furthermore, the use of 4D-LAQ parameters demonstrated good inter-observer reproducibility, indicating that this model has potential for application. In summary, we chose the LR model.

In conclusion, our study underscores the critical role of left atrial remodeling—particularly reduced LASr—in predicting MACE, linking these specific cardiac structural and functional alterations to the broader pathophysiological network of cardiorenal interactions. The prominent features in our predictive model, including elevated LVMI, reduced left atrial strain parameters (LASr, LAScd), and increased lnBNP, are hallmark indicators of pre-heart failure and hemodynamic stress. Such adverse cardiac remodeling rarely exists in isolation; rather, it frequently manifests alongside CKD and metabolic dysfunction, reflecting a synergistic multiorgan interplay where cardiac decline and renal deterioration mutually exacerbate one another. Therefore, our 4D-LAQ-based prediction model is designed to identify patients at high risk of MACE and promptly initiate cardiovascular monitoring for them. However, this study, as a single-center, small-sample retrospective study, has certain limitations and cannot guide treatment decisions. Clinical management should follow current guidelines and be combined with individualized assessment.

## Limitation

This study had several limitations. 1. As a single-center retrospective study, the sample size was relatively small. The EPV ratio was approximately 1.71, which is lower than the traditional threshold. Although the model did not show significant overfitting after multiple validations, external validation in larger-scale, multi-center cohorts is still necessary before its wide clinical application. 2.The data on the main causes of CKD and renal replacement therapy, as well as more baseline data such as blood potassium, HbA1c, and electrocardiogram features, have not been systematically recorded. Such data should be included in future multicenter prospective studies. 3. MACE is a composite endpoint, and each subtype (such as cardiac death and hospitalization for heart failure) may have different predictive factors. However, due to the small sample sizes of some subtypes, the predictive performance of this model for each subtype still requires further investigation. 4. This study aims to predict the occurrence of the first MACE, rather than to assess terminal events such as death. Future studies should continue to follow up after the first MACE, systematically determine all-cause mortality, and evaluate long-term outcomes while considering competing risks. 5. The end time of the group enrollment (January 2026) is quite close to the unified deadline (March 2026). Patients enrolled later had shorter follow-up periods, which may lead to an underestimation of the cumulative event rate. Future studies will require longer follow-up periods to verify this.

## Conclusions

4D-LAQ enables the assessment of left atrial function in patients with uremia and, when integrated with clinical indicators, supports the construction of a predictive model for MACE. LASr was an important predictor, and the nomogram based on these selected variables offers a practical reference for early cardiovascular risk stratification in this population group.

## Data Availability

The original contributions presented in the study are included in the article/**Supplementary Material**. Further inquiries can be directed to the corresponding authors.
