# Supplementary material for: Four-dimensional left atrial quantification for predicting major adverse cardiovascular events in patients with uremia: an interpretable LASSO-logistic model
Source: Front Endocrinol (Lausanne). 2026 Oct 30;17:1970519. doi: 10.3389/fendo.2026.1970519 (PMC13759426; doi:10.3389/fendo.2026.1970519)
Supplement: Supplementary file 1 [file Supplementaryfile1.docx]

**Supplementary Table 1:Abbreviation List**

| Noun | Explanation | Noun | Explanation |
| --- | --- | --- | --- |
| AUC | Area Under the Curve | LAScd | Left Atrial Strain during conduit phase |
| BNP | B‑type Natriuretic Peptide | LASct | Left Atrial Strain during contraction phase |
| BSA | Body Surface Area | LASr | Left Atrial Strain during reservoir phase |
| BUN | Blood Urea Nitrogen | LASSO | Least Absolute Shrinkage and Selection Operator |
| CKD | Chronic Kidney Disease | LAVI | Left Atrial Volume Index |
| CVD | Cardiovascular Disease | LAVmax | Left Atrial Maximum Volume |
| DBP | Diastolic Blood Pressure | LAVmin | Left Atrial Minimum Volume |
| DCA | Decision Curve Analysis | LAVpreA | Left Atrial Pre-A Volume |
| ESRD | End‑Stage Renal Disease | LDL | Low‑Density Lipoprotein |
| GFR | Glomerular Filtration Rate | LR | Logistic Regression |
| Hb | Hemoglobin | LVEDD | Left Ventricular End‑Diastolic Dimension |
| HDL | High‑Density Lipoprotein | LVEDV | Left Ventricular End‑Diastolic Volume |
| IVS | Interventricular Septum | LVEF | Left Ventricular Ejection Fraction |
| LAD | Left Atrial Diameter | LVPWT | Left Ventricular Posterior Wall Thickness |
| LAEF | Left Atrial Ejection Fraction | MACE | Major Adverse Cardiovascular Events |
| LAEV | Left Atrial Emptying Volume | ROC | Receiver Operating Characteristic |
| 4D-LAQ | The Four-dimensional Automatic Left Atrial Quantitative Technology | SBP | Systolic Blood Pressure |
| SHAP | SHapley Additive exPlanations | CV | cross-validation |

Supplementary Table 2: Hyperparameter search spaces and tuning strategy

| Model | Hyperparameter | Search space | Model | Hyperparameter | Search space |
| --- | --- | --- | --- | --- | --- |
| RF | n_estimators | 50, 100, 200, 300 | SGD | alpha | 0.0001, 0.001, 0.01, 0.1 |
|  | max_depth | 5, 10, 20, 50 |  | l1_ratio | 0.15, 0.25, 0.5, 0.75 |
|  | min_samples_split | 2, 5, 10, 20 |  | max_iter | 1000, 5000, 10000 |
|  | min_samples_leaf | 1, 2, 4, 8 |  | tol | 1e-5, 1e-4, 1e-3, 1e-2 |
|  | max_features | sqrt, log2, None |  | eta0 | 0.001, 0.01, 0.1, 1 |
|  | criterion | gini, entropy |  | loss | log_loss, modified_huber |
|  | bootstrap | True, False |  | penalty | None, l2, l1, elasticnet |
|  | class_weight | None, balanced, balanced_subsample |  | learning_rate | constant, optimal, invscaling, adaptive |
| SVM | C | 0.001, 0.01, 0.1, 1, 10, 100 | KNN | n_neighbors | 3, 5, 7, 9, 11, 13, 15 |
|  | degree | 2, 3, 4, 5 |  | leaf_size | 10, 30, 50, 100 |
|  | tol | 1e-3, 1e-4, 1e-5, 1e-6 |  | p | 1, 2, 3, 4 |
|  | max_iter | 1000, 5000, 10000 |  | weights | uniform, distance |
|  | kernel | linear, rbf, poly, sigmoid |  | algorithm | auto, ball_tree, kd_tree, brute |
|  | gamma | scale, auto |  | metric | euclidean, manhattan, minkowski, chebyshev |
|  | coef0 | 0, 1 | Adaboost | learning_rate | 0.1, 0.01, 0.001 |
|  | shrinking | True, False |  | n_estimators | 50, 100, 200 |
|  | class_weight | None, balanced |  | algorithm | SAMME, deprecated |
| LR | max_iter | 1000, 5000, 10000 |  | hidden_layer_sizes | (50,) (100,) (50,50) (100,100) (50,50,50) |
|  | C | 0.1, 1.0, 10.0 | MPL |  | (100,50,50) (50,100,50) (50,50,100) |
|  | penalty | 12 |  |  | (50,100,100) (100,50,100) (100,100,50) (100,100,100) |
|  | solver | lbfgs, saga |  | activation | relu, tanh |
|  | fit_intercept | TRUE |  | solver | adam, lbfgs |
|  | class_weight | None, balanced |  | alpha | 0.0001, 0.001, 0.01, 0.1 |
| Bayes | NA |  |  |  |  |

Supplementary Table 3: 5-fold stratified cross-validation mean ± standard deviation

| Model | accuracy | f1 | precision | recall | roc_auc |
| --- | --- | --- | --- | --- | --- |
| Logistic Regression | 0.7761 ± 0.0454 | 0.7058 ± 0.0750 | 0.7830 ± 0.0519 | 0.6497 ± 0.1062 | 0.8730 ± 0.0532 |

Supplementary Table 4:Nested 5×5 cross-validation performance

| Outer fold | AUC | Selected variables |
| --- | --- | --- |
| 1 | 0.842 | LASr, LDL, LVMI, Height, lnBNP, LAScd, LASr-c |
| 2 | 0.863 | LASr, LDL, LVMI, Height, lnBNP, LAScd, LASr-c |
| 3 | 0.885 | LASr, LDL, LVMI, Height, lnBNP, LAScd, LASr-c, LVPWT |
| 4 | 0.855 | LASr, LDL, LVMI, Height, lnBNP, LAScd, LASr-c |
| 5 | 0.833 | LASr, LDL, LVMI, lnBNP, LAScd, LASr-c |
| Mean ± SD | 0.856 ± 0.020 | - |
| Overall AUC (95% CI) | 0.844 (0.782–0.907) | - |

LVPWT was selected in only 1 out of the 5 outer compromises, indicating that its selection was unstable and did not receive consistent support from the data.Table note. Nested 5×5 cross-validation was performed within the training set (n = 156). In each outer fold, the outer test fold was held out completely; LASSO feature selection and hyperparameter tuning were performed only in the inner 5-fold loops. The selected variables were then used to fit a standard logistic regression model, which was evaluated on the held-out outer test fold. The overall AUC was calculated by pooling predictions from all five outer test folds. The original independent validation set (n = 67) was kept untouched and was not used in the nested cross-validation.

Supplementary Table 5:bootstrap internal validation

| Metric | Estimate | Lower_CI | Upper_CI |
| --- | --- | --- | --- |
| Apparent AUC | 0.894 | NA | NA |
| Mean Optimism | 0.025 | NA | NA |
| Optimism-corrected AUC | 0.869 | 0.855 | 0.891 |
| Calibration Slope | 1.000 | 0.834 | 1.418 |
| Brier Score | 0.132 | 0.125 | 0.134 |
| Scaled Brier Score | 0.458 | 0.449 | 0.484 |

Supplementary Table 6: calibration metrics

| **Model** | **Brier_Score** | **Log_Loss** | **MCE** | **Reliability_Slope** | **HL_p_value** |
| --- | --- | --- | --- | --- | --- |
| RF | 0.18 | 0.54 | 0.35 | 1.37 | 0.24 |
| SVM | 0.18 | 0.54 | 0.65 | 1.20 | 0.52 |
| LR(Model 3) | 0.16 | 0.49 | 0.29 | 1.24 | 0.36 |
| SGD | 0.19 | 0.57 | 0.31 | 1.05 | 0.32 |
| KNN | 0.20 | 0.57 | 0.44 | 1.28 | 0.27 |
| Adaboost | 0.16 | 0.50 | 0.39 | 1.69 | 0.06 |
| Bayes | 0.16 | 0.49 | 0.34 | 1.07 | 0.75 |
| MLP | 0.18 | 0.54 | 0.15 | 1.09 | 0.96 |
| Model 2 | 0.20 | 0.58 | 0.60 | 1.40 | 0.69 |
| Model 1 | 0.21 | 0.60 | 0.27 | 1.23 | 0.67 |

Brier Score, the mean squared difference between the predicted probabilities and the actual binary outcomes; Log Loss, the logarithmic loss that quantifies the divergence between predicted probabilities and true labels; MCE, the misclassification error rate, defined as the proportion of incorrect predictions; Reliability Slope, the slope of the calibration curve obtained by regressing observed event rates on predicted probabilities; HL p-value, the corresponding p-value of the Hosmer-Lemeshow test.

Supplementary Table 7:Decision curve analysis results of models

| Model | AUDC(95% CI) | Max_Net_Benefit |
| --- | --- | --- |
| LR-train | 0.215(0.213-0.215) | 0.41 |
| LR-test(Model 3) | 0.208(0.206-0.208) | 0.43 |
| Model 2 | 0.166(0.165-0.166) | 0.43 |
| Model 1 | 0.143(0.142-0.143) | 0.43 |

Supplementary Table 8:Variables retained by LASSO in the three prespecified models

| Model | Candidate variables, n | Retained variables, n |
| --- | --- | --- |
| Model 1 | Clinical(20) | 6(age,Height,lnBNP,Hb,LDL,gender) |
| Model 2 | clinical+echocardiography(26) | 8(age,Height,lnBNP,Hb,LDL,gender,LVPWT,LVMI) |
| Model 3 | clinical+echocardiography+4D-LAQ(38) | 7(LASr, Height, LVMI, LASr‑c, LAScd, lnBNP, and LDL) |

Supplementary Figure 1:model hyperparameter

The hyperparameter settings for each model (the Bays model has no hyperparameters)

Supplementary Figure 2:Bootstrap Calibration Curve

Supplementary Figure 2. Bootstrap calibration curve (B = 1000). The apparent , bias-corrected , and ideal calibration curves are shown. The bias-corrected curve closely approximated the ideal diagonal, indicating good calibration after optimism correction.
